# Silver Nanoparticles Biosynthesis, Characterization, Antimicrobial Activities, Applications, Cytotoxicity and Safety Issues: An Updated Review

**DOI:** 10.3390/nano11082086

**Published:** 2021-08-17

**Authors:** Deepak Bamal, Anoop Singh, Gaurav Chaudhary, Monu Kumar, Manjeet Singh, Neelam Rani, Poonam Mundlia, Anita R. Sehrawat

**Affiliations:** 1 Department of Botany, Maharshi Dayanand University, Rohtak 124001, India; deepakbamal55@gmail.com (D.B.); anoopsingh.rs.botany@mdurohtak.ac.in (A.S.); gauravjanghu10@gmail.com (G.C.); monubedi1995@gmail.com (M.K.); 2 Department of Genetics and Plant Breeding, Oilseeds Section, CCS Haryana Agricultural University, Hisar 125004, India; manjeetsingh125033@gmail.com; 3 Department of Botany and Plant Physiology, CCS Haryana Agricultural University, Hisar 125004, India; neelamkaswan009@gmail.com; 4 Department of Biochemistry, Punjab University, Chandigarh 160014, India; poonammundlia@gmail.com

**Keywords:** silver nanoparticles, biosynthesis, characterization, antimicrobial, SARS-CoV-2, cytotoxicity

## Abstract

Rapid advances in nanotechnology have led to its emergence as a tool for the development of green synthesized noble metal nanoparticles, especially silver nanoparticles (AgNPs), for applications in diverse fields such as human health, the environment and industry. The importance of AgNPs is because of their unique physicochemical and antimicrobial properties, with a myriad of activities that are applicable in various fields, including the pharmaceutical industry. Countries with high biodiversity require the collection and transformation of information about biological assets into processes, associations, methods and tools that must be combined with the sustainable utilization of biological diversity. Therefore, this review paper discusses the applicable studies of the biosynthesis of AgNPs and their antimicrobial activities towards microorganisms in different areas viz. medicine and agriculture. The confirmed antiviral properties of AgNPs promote their applicability for SARS-CoV-2 treatment, based on assimilating the virus’ activities with those of similar viruses via in vivo studies. In this review, an insight into the cytotoxicity and safety issues of AgNPs, along with their future prospects, is also provided.

## 1. Introduction

Nanotechnology represents a crucial turning point in the history of the universe. The endless desire and intelligence of humans have paved the way for groundbreaking inventions such as the internet, smartphones, rocket science, artificial intelligence and in vitro fertilization techniques, which also carry many moral issues; for this reason, nanotechnology is a field of science that was previously considered to be controversial. Overcoming these drawbacks, nanotechnology has emerged as an important tool in material science, which involves the fabrication, synthesis and manipulation of bulk molecules/particles in nanoscale dimensions. The fabricated objects, ranging in size from 1 to 100 nm, are called nanoparticles. The global applications of metal nanoparticles are due to their myriad aberrant properties. Being the smallest particle of a bulk object, nanoparticles manifest enhanced properties as more atoms are present on their surfaces, with less coordination than the bulk material [1]. Nanotechnology has unlocked new opportunities in diverse sectors such as food packaging, the environment, animal husbandry, agriculture and healthcare, making it one of the most fascinating industrial phenomena of the modern era. It is an emerging tool with which to address the challenges of making technology more sustainable and eco-friendly, with an increasing range of application [2].

Nanoparticles can be categorized into inorganic and organic classes. Inorganic nanoparticles mainly include metallic (Ag, Au), magnetic (Co, Ni) and semi-conductor (ZnO, CaSO_4_) types, while organic nanoparticles comprise carbon-based nanoparticles (carbon nanotubes, quantum dots) [3]. The exploitation of metal nanoparticles is beneficial due to their aberrant properties, such as their physical properties, reactivity and probable applications in diagnostics, drug delivery and antioxidant and antimicrobial studies. Aragon et al. (2015) studied the structural and hyperfine properties of Al-doped SnO_2_ nanoparticles manufactured via a polymer precursor approach [4]. Metal nanoparticles possess enhanced properties such as morphology, size and increased surface area in comparison to their bulk counterparts [1]. Due to the higher rate of silver commercialization, accounting for 55.4%, i.e., 313 out of 565, of all noble metal nanoparticle-based products available for consumers in the market, interest in AgNPs has grown rapidly [5]. Silver nanoparticles have been widely used for antimicrobial, anticoagulant, anticancer, orthopedic and thrombolytic purposes and in drug delivery, medical devices, sensing and diagnostics, etc. The importance of AgNPs is due to their catalytic activity, optical and thermal properties, chemical stability, thermal stability and antimicrobial activity [6]. A range of top-down and bottom-up approaches have been used to illustrate the tunable physicochemical and versatile functionality of AgNPs. Top-down approaches mainly involve evaporation–condensation processing methods, through which silver nanoparticles are constructed from large entities without atomic-level control. Meanwhile, bottom-up approaches mainly incorporate the electrochemical processing of metallic silver. This process begins with molecular components that are chemically assembled to build nanostructures using the principles of molecular recognition [7,8]. Particular attention has been paid to the inexpensive and environmentally friendly synthesis of AgNPs, which considers either the evaluation of reducing and antioxidant phytochemicals of plant origin, or a microorganism-mediated bioreduction mechanism. A range of AgNPs, including nanowires, pyramids, octahedral, tabular prisms and cubes, can be obtained by using many objects for the creation of NPs. AgNPs’ shape and size mainly depend on the response variables such as pH, temperature and concentration of Ag; meanwhile, in biological synthesis, they largely depend on the materials used for the production of AgNPs [9]. The concept of “green chemistry” has been expanding since the mid-1990s and researchers are seeking greener and more sustainable methods of AgNP synthesis. Green synthesis of AgNPs is simpler and easier, with minimal use of high-cost and potentially hazardous chemicals. Various types of bio-sources, such as bacteria, algae, fungi and plants, can be exploited, with their own numerous advantages and disadvantages for the green synthesis of AgNPs [10]. 

Considering the efficacy and applicability of AgNPs, this review first focuses on the green synthesis of AgNPs and the various experimental approaches used to investigate them. The antimicrobial activities of AgNPs, especially for SARS-CoV-2, along with their health and agricultural applications are also discussed. Finally, we discuss the cytotoxicity and safety issues associated with AgNPs and consider the potential of using AgNPs in future applications.

## 2. Green Synthesis of AgNPs

Extensive attention is being directed towards the synthesis of nanomaterials in the domain of applied physics, chemistry, catalysis and, most importantly, in diagnosis and therapeutic applications. The green synthesis of AgNPs has advantages over physical and chemical methods. The natural qualities of plant-derived secondary metabolites and single-step experimental installation stabilizes and reduces bulk silver to AgNPs. Biosynthesized approaches rely on the employment of eco-friendly chemicals or natural ingredients, which are less harmful to humans and the environment and are safe to use. During the chemical synthesis of AgNPs, the employment of expensive chemicals can carry health risks for workers and can be harmful to nature. Meanwhile, the physical approaches to nanoparticles synthesis incorporate abundant energy and force through energy-intensive processes that result in the high cost of the end-product and also environmental consequences. Nanobiotechnology, as derived from “green chemistry”, has huge possibilities for the development of novel and crucial end-products that benefit human health, the environment and industry [11]. It is estimated that one kg of raw silver costs approximately USD 14,000, while the synthesis of one kg of AgNPs would cost around USD 4 million [12]. Thus, the synthesis of silver nanoparticles via green approaches does not merely reduce the use of dangerous industrial chemicals but also the fallout from one-step manufacturing [13]. The biosynthesis of silver nanoparticles offers better control over the growth of nanoparticle crystals due to slow rate of synthesis and stabilization of the nanoparticles by dilution or steric hindrance. Depending upon the location at which the nanoparticle synthesis is carried out, the biosynthesis of nanoparticles can be divided into two main categories, namely either extracellular formation (nanoparticles form outside the cell) or intracellular formation (synthesized inside the cell). Intracellular formation of nanoparticles gives particles of appropriate shape and size, but they are not easy to separate from the reaction system in comparison to extracellular methods. Through extracellular synthesis, it is easier to understand the application of nanoparticles. Three important steps are required for the biosynthesis of AgNPs, i.e., bio-reduction of silver ions, controlled growth of the crystals to nanoparticles and stabilization of nanoparticles [14]. Microorganisms are of tremendous concern for nanoparticle synthesis; conversely, the process is susceptible to culture contagion, involves a time-consuming methodology and has less regulation over the nanoparticles’ size.

### 2.1. Bacterial-Mediated Synthesis of AgNPs

In the last decade, bacterial strains have been extensively used for the synthesis of inorganic nanomaterials (especially Se, Au, Ag) with appealing properties for the development of third-generation biosensors, promising diagnostic applications (cell-imaging and biolabeling, voltametric sensory devices) and for non-surface coating applications such as thin-film formation and annealing. Bacterial-mediated biosynthesized nanoparticles have also shown in vitro antimicrobial activity against pathogenic microbial strains and other properties such as antioxidant, anticoagulant, anticancer, anti-migration and antiproliferative [15]. Bacterial exposure to high metal ion concentrations reduces metal ions or forms complexes with metal ions for their survival. The metabolic pathway of some microorganisms is associated with metal ions, which, in turn, are required for their growth and are responsible for the bioconversion of metal ions into nanoparticles [14]. Various kinds of cellular transporters and oxidoreductase enzymes are involved, such as NADH-dependent nitrate reductase, and NADPH-dependent sulfite reductase flavoprotein subunit α and cysteine desulfhydrase are involved in intracellular and extracellular biocatalytic synthesis [15]. Bacterial species also have shown the ability to synthesize unique organic nanoparticles. A three-dimensional cellulose nanofibril network of aerobic acetic bacteria-like bacterial nanocellulose similar to genus *Gluconacetobacter*, the most competent bacteria for nanocellulose formulation, has previously been developed. Bacterial nanocellulose displays advanced purity, crystallinity and mechanical constancy in comparison to nanocrystalline cellulose and nanofibrillated cellulose [16]. Hence, bacterial nanocellulose is a nanomaterial that has attracted considerable interest for exploitation in biomedical applications (e.g., as antimicrobial agents, scaffolds for tissue engineering and for drug delivery systems) and on biosensor platforms [17]. Saeed and co-workers, in 2020, studied bacterial-mediated AgNPs and they showed noteworthy outcomes against human pathogens. The bacterial strains (*Escherichia coli* MF754138, *Exiguobacterium aurantiacumm* MF754139 and *Brevundimons diminuta* MF754140) were used to study secondary metabolite production. The bacterial strains used in the study exhibited numerous possibilities as antimicrobial agents against methicillin-resistant *Staphylococcus aureus* (MRSA) and numerous other multidrug-resistant (MDR) bacteria with a 10–28 mm zone of inhibition. The study reported an eco-friendly method for the creation of AgNPs, which will be helpful to control the nosocomial infections caused by MRSA and other human pathogens [18]. Huq reported a straightforward and eco-friendly process of AgNP formulations using *Lysinbacillus xylanilyticus* MAHUQ-40 to target antibiotic-resistant human microbes *Vibrio parahaemolyticus* and *Salmonella typhimurium.* Confirmation and characterization of AgNPs were performed via UV–visible spectroscopy, X-ray diffraction (XRD), Fourier-transform infrared (FTIR), field emission-transmission electron microscopy (FE-TEM) and dynamic light scattering (DLS) [19]. Ma et al. (2018) investigated AgNPs extracellularly biosynthesized with *Streptomyces coelicoflavus* KS-3. The AgNPs were spherical or nearly spherical in shape, followed by a small amount of shortened triangular, quadrangular and hexagonal particles extending from 2.33 to 91.3 nm in diameter. Moreover, a similar study on the cytotoxic effects of AgNPs indicated that AgNPs exhibited a good cytotoxic outcome in a dose-dependent approach against HTB-182 and A549 cells ranging in concentration from 1 to 50 µg/mL [20]. The bacterial strains used for the biosynthesis of AgNPs are summarized in Table 1.

### 2.2. Algal-Mediated Synthesis of AgNPs

Algae have long been exploited in food, feed, cosmetics, fertilizers, additives and pharmaceuticals. Recently, research has focused on the algal-mediated biosynthesis of nanoparticles. Algae are easy to cultivate, scalable, have rapid growth and are an excellent source of secondary metabolites. Due to these facts, interest in algal-mediated nanoparticle synthesis is increasing rapidly [29]. Algae, varying in size from microscopic (picoplankton) to macroscopic (rhodophyta), have been used for the synthesis of AgNPs. Algal strains such as *Tetraselmis kochinensis*, *Desmodesmus* and *Scenedesmus* have been adopted for the biological synthesis of novel metal nanoparticles. These NPs are prominently used for antimicrobial purposes and in drug delivery, electronics, catalysis and other biomedical techniques [15]. An alga hyper-accumulates heavy metals and possesses the ability to convert them into new, variable forms. Due to these appealing attributes, alga have been used to develop a range of nanomaterials. The mechanism of the algal-mediated biosynthesis of nanoparticles is characterized by the ability to control the dimensions and the phenomena of nucleation, stabilization of nanoparticles’ structure and regulation by reducing agents [30], biomolecules [31] and enzymes [32]. Merin et al. (2010) formulated AgNPs by using marine algae as a reducing and stabilizing agent [33]. A biomineralized silica cell wall called a frustule is present in the unicellular microalgae (diatoms) and forms a periodic and hierarchical 3D porous micro-nanostructure of diverse configurations. The usual functions of frustules are mechanical protection, DNA shielding from UV, biological protection and filtration and optimization of light harvesting [34,35]. Diatom frustules possess several advantages over silica materials (e.g., MCM-4), including higher biocompatibility, ease of purification and reduced toxicity. The secondary metabolites produced from algal species have been used to stabilize, cap and reduce the main metal to form metal, metal oxide or bimetallic nanoparticles. Among the diverse range of algae, red (Rhodophyceae), brown (Phaeophyceae), blue-green (Cyanophyceae), micro- and macro-green algae are the most extensively explored algae for the biosynthesis of nanoparticles to date. Till now, over 20 different green micro-algal species have been utilized for the formulation of silver nanoparticles. Algal-mediated biosynthesized AgNPs, when analyzed via different microscopic and spectroscopic techniques (SEM, XRD, FTIR, EDX, DLS), all exhibit interesting and variable physico-chemical attributes [30,36,37]. 

Due to the presence of various precious compounds that are responsible for the reduction and capping of nanoparticles, green macro-algae are considered bio-factories for the creation of metallic NPs. *Ulva fasciata* is the most common green alga used to form nanosized colloids [38]. *Chaetomorpha linum* is an important macro-algal green seaweed species used to formulate silver nanoparticles by prompting the reduction of Ag^+^ to Ag^0^ with the assistance of terpenoids, peptides and flavonoids. *C. linum* is widely acknowledged for its biological importance in the control of nutrients accessibility to its habitat [29]. Ulagesan et al. (2021) studied the biogenic preparation of AgNPs using an aqueous extract from marine red algae (*Pyropia yezoensis)*. Spherical-shaped silver nanoparticles were confirmed, with an average crystalline size of 20–22 nm. Gram-positive and Gram-negative bacterial strains were used to study the antibacterial properties of AgNPs. The growth of *Pseudomonas aeruginosa* was reduced at concentration of 200 and 400 µg/mL by using *Pyropia yezoensis* AgNPs [39]. Algal species that have been extensively used in the past for the biosynthesis of AgNPs are listed in Table 2.

### 2.3. Fungal-Mediated Synthesis of AgNPs

Fungi have tremendous potential for the creation of many compounds. Around 6400 bioactive compounds are acknowledged to be formed by microscopic filamentous or non-filamentous fungal species [46]. Owing to heavy metal acceptance and the ability to internalize and bioaccumulate metals, fungal species are widely used as stabilizing and reducing agents. Furthermore, fungi can generate NPs with controlled size and morphology at a large scale in “nanofactories” [47,48]. The fungal-biosynthesized nanoparticles can be intracellular or extracellular. Although many studies have been conducted on the biosynthesis of AgNPs using fungi, the exact mechanisms have not yet been fully elucidated. It has been suggested that the extracellular formation of NPs takes place as the enzymes in the fungal filtrate act to reduce silver ions to elemental silver (Ag^0^) at a nanometric scale [49,50]. The enzymes most involved in the biosynthesis of metallic nanoparticles are NADH and NADH-dependent nitrate reductase [51,52]. The synthesis of endophytic AgNPs (EFNps) was performed using the aqueous extract of endophytic fungi (*Lasiodiplodia theobromae*) sheltered by *Cinnamomum zeylanicum*. Antimicrobial evaluation—for instance, minimum inhibitory concentration (MIC), minimum bacterial concentration (MBC), agar well diffusion, pyocyanin, antibiofilm and time kill curve, was performed against *P. aeruginosa* ATCC (27853) and an antibiotic-resistant clinical strain. Results suggested that EFNps could be a potential substitute for antibiotics to cope with the infections caused by *P. aeruginosa*. The fungal-mediated biosynthesis of AgNPs is a sanitary, safe, eco-friendly, reliable, inexpensive and green approach that can be used for a wide array of applications in everyday living [53]. Ammar et al. (2021) aimed for the biosynthesis, characterization and biomedical application of AgNPs via yeast metabolites. AgNPsK and AgNPsU were synthesized from the yeast strains *Pichia kudriavzevii* HA-NY1 and *Saccharomyces uvarum* HA-NY3, respectively. Highly significant inhibitory activity was shown by AgNPs hostile to Gram-positive bacteria (*Staphylococcus aureus* ATCC29213 and *Bacillus subtilis* ATCC6633) and Gram-negative bacteria (*Fusarium oxysporium* NRC21, *Pseudomonas aeruginosa* ATCC27953 and *Candida tropicalis* ATCC750). This study also showed the significant anticancer activity of both AgNPsK and AgNPsU against PC3 (prostate cell line), with IC50 values of 0.57, 0.50 µg mL^−1^ respectively, and HCT-116 (colon cell line), with IC50 values of 0.29, 0.24 µg mL^−1^, respectively. AgNPs were also found to be safe for the gastric profile because no ulcerogenic effects were detected in rats’ stomachs [54]. Another researcher synthesized AgNPs using *Punica granatum* extract as a reducing and stabilizing agent. A cubic crystalline structure with face-centered AgNPs was determined by using the XRD technique. The study revealed that AgNPs synthesized via *Punica granatum* extract may have a possible use in the fields of nanobiotechnology, nanomedicine and nanobiosensors [55]. The most recent studies of AgNP biosynthesis using fungal strains, along with their key attributes, are summarized in Table 3.

### 2.4. Plant-Mediated Synthesis of AgNPs

The plant-mediated biosynthesis of AgNPs is a straightforward practice, highly effective and with short reaction times. Due to the presence of numerous metabolites (phenols, ketones, proteins, aldehydes, amides, carboxylic acids), plants have the ability to reduce and stabilize nanoparticles. In the biosynthesis of AgNPs, almost every plant part, i.e., leaves, seeds, roots and flowers, has been used for the mining of active ingredients [10]. From close observations in recent studies on the biosynthesis of AgNPs, it can be inferred that interest is shifting significantly towards the exploitaion of medicinal flora for nanoparticle synthesis. Because of the occurrence of abundant reducing components (H^+^), the green leaves of medicinal plants possess the prime capacity to reduce and stabilize AgNPs [62]. Various kinds of natural compounds with proven actions against bacteria, microbes, cancerous cells and neurodegenerative disorders have been extracted from medicinal plants. Thus, the incorporation of medicinal plants into biosynthesis development could move beyond simply a green chemistry approach, enhancing the biological properties of nanoparticles [10]. In this regard, several studies have reported on the synthesis of AgNPs from medicinal plants for various applications. Jain and Mehata (2017) reported the biosynthesis of AgNPs using *Ocimum sanctum* leaf extract due to its exceptional antibiotic and germicidal properties. The reduction of AgNPs took place due the presence of hydroxyl and ketone groups in tulsi leaf extract and improved antibacterial activities were observed against *E. coli* [63]. *Azadirachta indica* was exploited for the biosynthesis of AgNPs and antibacterial activities against *S. aureus* and *E. coli* were observed [64]. *Teucrium polium* and *Ocimumbasilicum* were evaluated for AgNP formation, with potential antibacterial, antitumor and antioxidant activities. Biosynthesized AgNPs were also employed to study their cytotoxic activities against the HEP G2 cell line [65]. Ghramh et al. (2020) formulated spherical AgNPs of 40–45 nm using the ethanolic extract of *Ruta graveolens* and studied the different biological activities, immune modulation, anticancer and insecticidal potential in the extracts. The extract with AgNPs showed insecticidal activity against *Culex pipiens* [66]. Palithya et al. (2021) demonstrated the high negative zeta potential (−26.0 mV) value of *Decaschistia crotonifolia* biosynthesized Dc-AgNPs [67]. Singh et al. (2021) reported the antimicrobial activities of *Carissa carandas* biosynthesized AgNPs against human pathogenic bacteria [68]. Plant-mediated biosynthesized AgNPs along with their bioactivities are listed in Table 4.

## 3. Characterization of AgNPs

Characterization is a vital step that involves determining the size, shape, surface area, morphology, charge, dispersity and surface chemistry of nanoparticles. There is a need for concurrent development involving a wide range of techniques for a complete understanding of the system and the characterization of synthesized nanoparticles using analytical techniques such as UV–visible spectroscopy, Fourier-transform infrared spectroscopy (FTIR), X-ray diffraction (XRD), X-ray photoelectron spectroscopy (XPS), scanning electron microscopy (SEM), transmission electron microscopy (TEM) and dynamic light scattering (DLS). AgNP formation is verified by the appearance of a yellowish-brown color when AgNO_3_ is added to the culture filtrate. Confirmation can be achieved by using a culture filtrate without AgNO_3_ as a control. This occurs due to the excitation of plasmons on the surface of the atomic lattice of nanosized materials by light; this color change is termed surface plasmon resonance [14]. The pioneering work of Ritchie in 1950 [106] widely recognized the applications of surface plasmons in the field of surface science [107]. A strong absorption band appears in the UV–visible spectra of noble metal nanoparticles, which is usually absent in their bulk form. This is because of the collective oscillation of the conductive electrons with incident photons, and it is termed localized surface plasmon resonance (LSPR) [108]. The interaction of coherent localized oscillations with a resonant frequency largely depends on the size, geometry, dielectric environment, composition and particle–particle separation distance of NPs [109]. Because of the presence of *d-d* transitions exhibiting LSPR, noble metals are used for the formulation of NPs [110]. NPs exhibit a molar extinction coefficient of approximately 10^11^ M^−1^ cm^−1^ for absorption undergoing LSPR. However, distinct from fluorophores, plasmonic NPs do not photobleach or blink and therefore serve as intense and robust labels for immunoassays, biosensors, surface-enhanced spectroscopies and cellular imaging [111,112,113]. There is only one SPR absorption peak in the case of AgNPs, while silver nanorods (AgNRs) show two SPR absorption peaks, i.e., transverse and longitudinal [114]. Cytotoxicity and chemical degradation are the two major drawbacks of AgNPs. To overcome these drawbacks, scientists have developed a new synthetic approach for highly monodisperse polymer-coated AgNRs, which are noncytotoxic and protected against external stimuli such as heat, light and oxidation [115]. A different method of sample preparation is required for each of the characterization techniques. For ease of understanding, the fundamentals of the key approaches employed for the characterization of AgNPs are described in the following subsections. 

### 3.1. UV–Visible Spectroscopy

In support of the primary characterization of NPs, UV–visible spectroscopy is an extremely practical and reliable technology. UV–visible spectroscopy assesses the synthesis and stability of AgNPs. The distinctive optical properties of AgNPs make them very interactive with particular wavelengths of light [116]. Because of the phenomenon of surface plasmon resonance (SPR), AgNPs display good absorption in the visible spectrum, with a maximum in the range of 400–500 nm. The resonant collective oscillations of conductive electrons along the transverse direction of the electromagnetic field cause SPR in the UV–visible region of the spectrum. Nanoparticles’ shape and temperature, the chemical surroundings and the dielectric constant of the medium influence the SPR band strength and bandwidth [117]. Observations of SPR peak intensity determine the sizes of various nanoparticles ranging from 2 to 100 nm. The stability of biosynthesized AgNPs was investigated for more than one year and an SPR peak of a similar wavelength was observed by means of UV–visible spectroscopy [118,119]. In this way, UV–visible spectroscopy is an efficient tool to characterize the AgNP structure kinetics and the final colloidal stability. Haiss et al. (2007) theoretically and experimentally determined the optical properties of AuNPs in aqueous solution using UV–vis spectra and established a correlation between the extinction efficiency (*Q_ext_*) and *d*, which determines the particle concentrations (*c*) [120]. Albert et al. (2021) successfully performed characterization studies of ZnSO_4_-doped CeO_2_ nanoparticles using UV–visible spectra and calculated the band value using the Tauc plot method [121].

### 3.2. Fourier-Transform Infrared (FTIR) Spectroscopy

FTIR is a spectroscopic technique used to accurately and reproducibly estimate the signal-to-noise ratio. Minute absorbance varying on the order of 10^−3^ can be detected via FTIR spectroscopy [116]. The occurrence of functional groups in the biosynthesized extracts (plants or microorganisms), which are responsible for the reduction and stabilization of silver ions, can be efficiently determined with the help of the FTIR technique. FTIR is used to investigate the surface chemistry of metal nanoparticles [122]. During FTIR analysis, IR radiation is passed through the sample, resulting in some IR radiation being absorbed by the sample while some passes through it. The resulting spectrum shows absorbance and transmittance by creating a molecular imprint of the sample that represents the identity of the sample [123]. Consequently, FTIR is an appropriate, useful, cost-effective, non-invasive and modest technique to investigate the function of biomolecules in the reduction of AgNO_3_ to Ag. Bhambure et al. (2009) studied the characterization and stability of freeze-dried powdered AuNPs diluted with potassium bromide at a ratio of 1:1000 using the FTIR pattern, which revealed the strong interaction of AuNPs with the structural proteins of *Aspergillus niger* NCIM 616 [124]. Alamdari et al. (2020) performed FTIR analysis for the identification of functional groups (flavonoids, anthocyanins and cyaniding-3-glucoside) in *Sambucus ebulus* extract that contribute to the mechanism of bonding with ZnO NPs [125]. 

### 3.3. X-ray Diffraction (XRD)

For the assessment of both the molecular and crystal configuration of a compound, XRD is a very valuable analytical technique [126,127]. XRD determines particle sizes, the degree of crystallinity, isomorphous substitutions and the qualitative and quantitative resolution of various chemical compounds. During XRD analysis, an X-ray beam is projected onto the crystal and scattered by the atoms, which leads to the creation of diffraction patterns. The interference of scattered X-rays can be used according to Bragg’s law to identify the several characteristics of the crystal or polycrystalline material [123]. XRD confirms the crystalline nature of the NPs by defining the oxidation state of the particles as a function of time, and the measurements of XRD are generally calculated in Angstroms (1 Å = 0.1 nm). Even though XRD has a number of advantages, it also has some drawbacks, including obscurity in mounting the crystals, single conformation/binding state and low intensity of diffracted X-rays [128,129].

### 3.4. Scanning Electron Microscopy (SEM)

The field of nanotechnology and nanoscience has provided a driving force for the improvement of a range of high-resolution microscopic techniques. These microscopic techniques use a shaft of light of extremely energetic electrons to probe objects [130]. A scanning electron microscope is a surface imaging tool used for analyzing the diverse particle sizes, size distributions, nanomaterial shapes and the surface morphology of synthesized particles at the nanoscale [131]. The combination of SEM with energy-dispersive X-ray spectroscopy (EDX) can be used to determine the elemental composition of an AgNP sample [132]. The X-rays emanated by the sample are detected using the EDX technique and an EDX ray detector quantifies the comparative abundance of discharged X-rays vs. their energy [133]. The main advantage of SEM is that it can deliver valuable information regarding the purity and extent of particle aggregation, but the drawback is that it is not suitable to examine the interior structure of the sample. 

### 3.5. Transmission Electron Microscopy (TEM)

TEM is used for the quantitative measurement of the particle size, morphology and distribution of nanomaterials. TEM projects an electron beam onto a sample and forms an image on a photographic plate [134]. The advantage of TEM over SEM is that it can provide an improved resolution and further critical dimensions [135,136]. The drawbacks of TEM include the need for a high vacuum and a large sample section. In TEM analysis, the sample preparation is laborious, but it tremendously useful to attain refined-quality images [116].

### 3.6. Dynamic Light Scattering (DLS)

Dynamic light scattering (DLS) is an important, fast, easy and non-destructive tool for characterizing nanoparticles that is used to grade the particle size at the micro- and nanometer regimes. During DLS, a laser passes through a colloidal solution, which scatters light at different intensities due to the Brownian motion, and the size is graded using the Stokes–Einstein relationship. The hydrodynamic diameter (the diameter of the hypothetical nonporous sphere that diffuses at the same rate as the particles being characterized) can be calculated from the time dependence of the scattering intensity measurements. The presence of an electrical double layer absorbed on the surfaces of nanoparticles and the capping agent/stabilizer usually affects the hydrodynamic diameter of the nanoparticles [105,137,138,139,140,141]. The mono-exponential equation is used for samples with purely monodisperse particles in DLS, where the fluctuation of intensity in scattered light is correlated against short decay intervals (τ) and the intensity autocorrelation function (ACF) is calculated [142].

G(τ) = 1 + b.e^−2D^_t_^q2τ^

Here, b = constant dependent upon the instrument and settings of optics, D_t_ = translational diffusion coefficient and q = scattering vector.

Tomaszewska et al. (2013) studied the size distribution of chemically synthesized polydisperse silver nanoparticle colloids using DLS. The result revealed that several percent of the volume content of large NPs could screen completely the presence of smaller ones [139].

## 4. Antimicrobial Activities of AgNPs

The most significant medical achievement of the 20th century was antibiotics, as this discovery played a decisive role in treating patients with cancer or diabetes, and those experiencing microbial infections and surgical complications [143]. Regrettably, this achievement led to the emergence of antimicrobial resistance (AMR). The development of multidrug-resistant microorganisms (MDRM) has occurred due to the excessive use of antimicrobial agents against harmful pathogens [144]. Pathogenic resistance is a serious problem encountered by pharmacists and healthcare professionals. An individual septic with multidrug-resistant bacteria (MDR) cannot simply be cured since they need to be treated with a wide range of antibiotics [145]. According to the World Health Organization (WHO), drug-resistant pathogens cause a high mortality rate during disease pandemics [146]. Therefore, the advancement and alteration of antimicrobial compounds with improved uptake has been a major interest in recent years. During the last few decades, research has been concerned with the biomedical applications of metallic nanoparticles resulting from metals such as Ag, Au, Cu, Pt, etc. Among them, AgNPs have attracted major consideration due to their unique antimicrobial properties. The exact mechanism behind the antimicrobial action of AgNPs has not been illustrated yet, but through recent research studies, it has been found that, due to their small size, AgNPs can easily penetrate the microbial cell wall and generate reactive oxygen species (ROS) and free radicals, which leads to apoptosis [147]. The development of nanotherapeutics has received a great deal of attention, particularly regarding AgNPs because of their broad-spectrum oligodynamic properties. Dating back to 1881, the first evidence of using silver in medicine was reported to treat eye infections in neonates and, soon after, in 1901, for internal antisepsis. Currently, drugs such as silver nitrate and silver sulfadiazine are frequently used to treat wounds and dermal burns and to eradicate warts [148]. Feng et al. (2000) used electron microscopy and X-ray microanalysis to perform a mechanistic study of the antibacterial action of Ag^+^ on *E. coli* and *Staphylococcus aureus*. The results of electron microscopy showed an electron-light region in the center of the cells and many small, electron-dense granules either surrounding the cell wall or deposited inside the cells. Meanwhile, X-ray microanalysis revealed the existence of Ag elements and sulfur in electron-dense granules and cytoplasm. The study also suggested that DNA lost its replication potential and the protein became activated after Ag^+^ treatment [149]. During World War I, silver was the most used material in to treat infections among soldiers [150]. The antimicrobial applications of AuNPs are related to their non-toxicity, ease of detection, polyvalent effects, high capacity for functionalization and photothermal properties [151]. Because of their biocompatibility, functionalization and covalent bonding to low-molecular-weight chitosan, AuNPs can also be used as a vehicle for drug and vaccine delivery [152]. Usman et al. (2013) tested the antimicrobial activities of Cu–chitosan NPs (2–350 nm) against *S. aureus*, *B. subtilis*, *C. albicans*, *P. aeruginosa* and *Salmonella choleraesuis* [153]. Cho et al. (2004) studied the antimicrobial activities of Ag and Pt NPs against *S. aureus* and *E. coli.* using sodium dodecyl sulfate (SDS) and poly-N-vinyl-2-pyrrolidone (PVP) as stabilizing agents [154]. This section of the review is focused on the main achievements and the mechanisms of AgNPs as nanocarriers for the inhibition of a variety of microbial infections. 

### 4.1. Antibacterial Action of AgNPs

**Figure 1:**
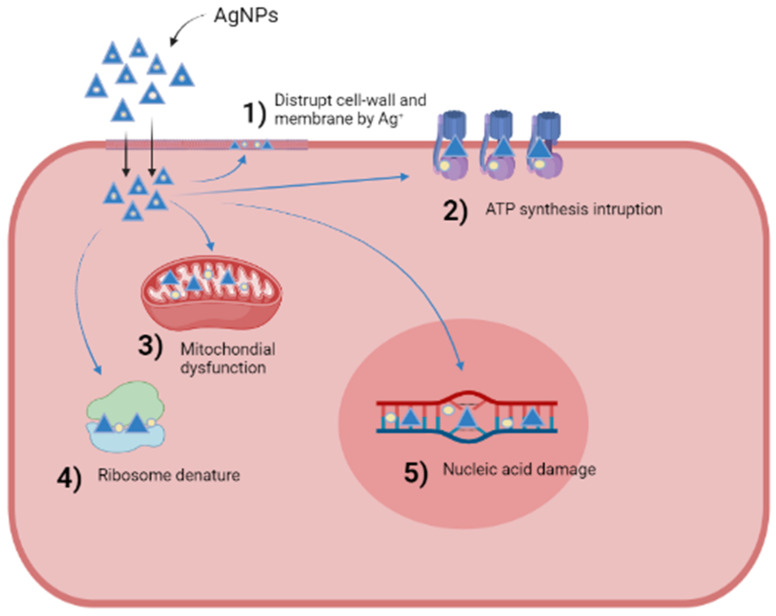
The mechanism of antibacterial action of AgNPs illustrated as (1) disruption of cell wall and membrane by silver ions released from AgNPs; (2) AgNPs inhibiting ATP synthesis; (3) mitochondrial dysfunction caused by Ag+ released from AgNPs; (4) ribosomal degradation by AgNPs; (5) AgNPs damaging nucleic acid by incorporating Ag+.

Bacterial resistance to antibiotics is a major health issue with a massive impact globally. In the last few decades, pharma-industries have focused their concern on developing novel antibiotics with better capability to target bacterial diseases [155]. Nanoparticles have become very useful against bacterial infections considering their large surface volume area and high synergy arising from their multivalent interactions. AgNPs are the most extensively used antibacterial nanoagent because of their wide-ranging antimicrobial effectiveness against several bacteria [156]. AgNPs intermingle via the bacterial cell envelope, yet the prime cellular target remains unknown. The combined effects of AgNPs along with antibiotics lead to increased antibacterial activity against drug-resistant bacteria [157]. The combined uptake of AgNPs and popular antibiotics such as kanamycin, tetracycline, enoxacin and neomycin led to the suppression of multiple-drug-resistant *S. typhimurium* bacterial growth; however, this synergistic effect is not observed in the case of penicillin and ampicillin [158]. The antibacterial activity of AgNPs along with other common antibiotics such as erythromycin, vancomycin, amoxicillin, ciprofloxacin, streptomycin, tetracycline and gentamicin is improved against *E. coli* and *S. aureus* [159], while kanamycin, ampicillin and chloramphenicol showed synergistic effects against various bacterial strains including *St. mutans*, *St. aureus*, *Ent. faecium* and *E. coli* [160]. Nanomaterials possess superior antibacterial potency against Gram-positive bacteria in comparison to Gram-negative bacteria. AgNPs can constantly liberate Ag^0^, which may be considered a means of killing microbes. The approaches that illustrate the mechanism of antibacterial action of AgNPs are summarized in Figure 1. The adherence of silver ions to the cytoplasmic membrane and cell wall occurs because of the electrostatic affinity of Ag^0^ towards sulfur proteins. This leads to the disruption of the bacterial envelope by enhancing the permeability of the cytoplasmic membrane [161]. The uptake of Ag^0^ into the cells results in the deactivation of respiratory enzymes, the formation of ROS and interruption of ATP production [162]. ROS can play a prime role in the processes of DNA modification and cell membrane disruption. The interaction of Ag^0^ with the sulfur and phosphorus components of DNA results in DNA modification. Likewise, Ag^0^ can hinder the formulation of proteins by denaturing ribosomes in the cytoplasm [163].

Silver nanoparticles can also kill bacteria themselves, without the release of silver ions. After the attachment of AgNPs to the cell surface, they accumulate in the cell wall pits, which results in cell membrane disruption [164]. Likewise, AgNPs also disrupt the bacterial signal transduction by dephosphorylate tyrosine residues on the peptide substrates, which causes the termination of cell multiplication and apoptosis [165]. The antibacterial action of AgNPs also depends on the dissolution status of AgNPs in exposure media. The synthetic and processing factors, such as intrinsic AgNP characteristics (shape, size, capping agent) and surrounding media (organic and inorganic components), directly affect the dissolution efficacy of AgNPs [166,167,168,169]. The thick cellular wall of Gram-positive bacteria may reduce the penetration of AgNPs into their cells; this is why Gram-negative bacteria are more susceptible to AgNPs [170]. The biofilm formation in the oral environment protects bacteria from both Ag^0^ and AgNPs by hindering their transport. The bioavailability and mobility of AgNPs in the biofilm is determined by the AgNPs’ diffusion coefficients [171,172,173]. Ivan Sondi and B.S. Sondi (2004) also studied the biocidal activities of AgNPs against *E. coli* and confirmed the “pit” formation in the cell wall of this model Gram-negative bacterium. A significant increase in permeability occurred due to the accumulation of AgNPs in the bacterial membrane, resulting in cell death [174].

### 4.2. Antifungal Action of AgNPs

Since the primeval era, fungal infections have contributed considerably to the escalating morbidity and mortality. Studies have found that outbreaks caused by pathogenic fungi can be controlled by exploiting the fungicidal or fungistatic activity of nanoparticles [175]. Biosynthesized AgNPs stabilized with sodium dodecyl sulfate (SDS) exhibit good antifungal activity compared to fluconazole and are hostile towards phytopathogens such as *Aspergilus niger*, *Trichophyton mentagrophytes*, *Fusarium semitectum*, *Candida glabrata*, *Issatchenkia orientalis*, *Phoma glomerata*, *Candida albicans* and *Phoma herbarum* [176]. 

Panacek et al. (2009) demonstrated the potent antifungal activity of AgNPs stabilized with surfactants of size 25 nm, which are hostile towards four *Candida* strains, with an MIC value extending from 0.21 to 1.69 mg/L [177]. The antifungal activity of corn extract biosynthesized AgNPs hostile to the phytopathogenic fungus *Phomopsis vexans* was demonstrated by reducing the expansion of mycelium by 30–40% in Potato Dextrose Agar (PDA) medium [178]. Elgorban et al. (2016) analyzed the antifungal action of AgNPs against plant pathogenic fungi *Rhizoctonia solani* infecting cotton plants [179]. Similarly, the antifungal activity of Ag ions and AgNPs hostile to two plant pathogenic fungi (*Bipolaris sorokiniana* and *Magnaporthe grisea*) was reported [180]. 

The following mechanistic pathways are considered responsible for the antifungal activity of AgNPs: (i) fungal cells easily uptake AgNPs due to their small size, which leads to the disturbance of fungal cell walls; (ii) AgNPs perform as a source of Ag^+^ ions that stops DNA replication and ATP synthesis, by hydroxyl radical and ROS formulation. Because of this, the biochemical cycle of fungal cells is stopped, inducing fungal cell death. Owing the strong tendency of Ag^+^ for the thiol groups of the cysteine protein in fungal cells, Ag^+^ ions exhibit anticandidal activity through the inactivation of ATP synthesis and enzyme function, leading to cell death [181]. 

### 4.3. Antiviral Action of AgNPs 

Currently, viruses are recognized as one of the most significant causative agents of human disease. Regardless of their evident structural simplicity, viruses pose a massive threat in the face of perilous diseases, such as the Spanish flu, HIV, Ebola, Marburg virus, and, lastly, the 2020 pandemic caused by COVID-19 [9]. Viral infections pose major challenges to global health, particularly given the fact that the appearance of resistant viral strains and the deleterious side effects associated with long-term use persist to slow the adoption of effectual antiviral therapies. This makes it crucial to develop safe and efficient alternatives to prevailing antiviral drugs. The recent insurgence of COVID-19, which has developed resistance towards existing antiviral drugs, has led researchers to search for new antiviral agents. The SARS-CoV-2 pandemic began in December 2019 and has killed more than 3.2 million people worldwide as of May 2021 [182]. The pathogenic nature of viruses depends on their attachment and infiltration into the host cells via the binding of viral surface constituents with ligands and proteins on the cell membrane. Thus, the preeminent approach in developing new antiviral drugs is to prevent such bindings. In the current situation, metal nanoparticles have emerged as novel antiviral agents because of their exceptional physical and chemical properties; AgNPs have become important candidates as antiviral agents. AgNPs wide range of mechanisms of attack towards their targets can reduce the microbial resistance to these nanoparticles [183]. Table 5 summarizes the most significant studies on AgNPs’ antiviral activity towards different viruses. However, details of the exact mechanisms of their action or interactions are limited so far.

**Figure 2:**
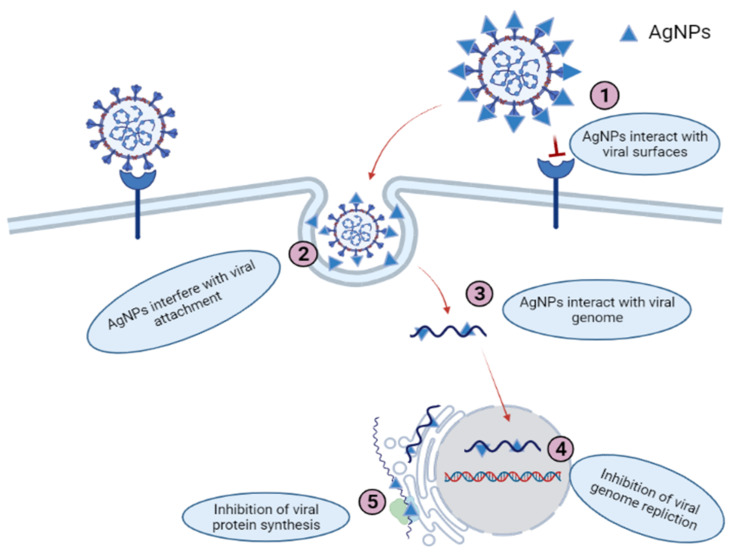
The possible mechanism of AgNPs’ antiviral efficacy.

The complexity of the virus structure may contribute to the limited knowledge of the mechanism of interference of nanoparticles with viruses. There are two possible methods by which AgNPs exert their antiviral activities, which are as follows: (i) AgNPs bind to the outer coating of proteins, thus suppressing the attachment of the virus to cell receptors; (ii) AgNPs bind to nucleic acid (DNA/RNA) and inhibit the replication or proliferation of the virus inside the host cells [183]. It is evident that AgNPs can alter the structure of surface proteins, thus reducing their detection and adhesion to the host receptor. Figure 2 shows the mechanism of AgNPs’ antiviral efficacy. AgNPs prevent the commencement of transmitted gastroenteritis virus (TGEV) by binding to a surface protein, S-glycoprotein [184]. Furthermore, Sharma et al. (2019) focused on the biological creation of AgNPs from medicinal plants (*Tinospora cordifolia*, *Andrographis paniculata* and *Phyllanthus niruri*) and evaluated their antiviral properties against chikungunya virus [208].

A crucial characteristic of nanoparticles in relation to Coronavirus (CoV) is that they are able to compete with viral binding to a receptor on the surface of a host cell. Moreover, the penetration of CoV into host cells is mediated by ACE2 receptors, particularly in the case of SARS-CoV and SARS-CoV-2 [209]. Therefore, blocking and/or lowering ACE2 levels could help to fight CoV infections as well as generate antibodies against ACE2. However, on the other hand, increased production of vasodilating angiotensin 1–7 was found to occur due to the protective action of ACE2 against viral lung damage after infection [210]. Hence, obviating COVID-19 in the host may be more helpful than fighting the virus after infection. Research studies on HIV have shown the attachment of AgNPs to disulfide bonds of the CD-4 binding domain of the glycoprotein 120 (gp120) surface proteins [201]. Similarly, AgNPs can disrupt the disulfide bonding on ACE2 and spike protein and exert their antiviral efficacy against SARS-CoV-2. The interaction of AgNPs with the ssRNA of Coronavirus improves the antiviral activity of AgNPs. The most effective antiviral efficacy of AgNPs occurs in those with a diameter of 10 nm. This was confirmed by an immunofluorescence study involving 10 nm AgNPs capped with polyvinylpyrrolidone, which completely inhibited SARS-CoV-2’s activities in comparison to 100 nm AgNPs, which did not [211]. AgNPs also showed their efficacy against key determinant proteins of pathogenicity, namely hemagglutinin (H) and neuraminidase (N). The combination of AgNPs with antiviral drugs such as zanamivir, osltamivir, FluPed and amantadine results in the production of ROS, which reduces the destruction of MDFK cells and reduces H and N proteins’ action in healthy cells [197,212,213]. Thus, it can be concluded that AgNPs’ potent effects for SARS-Co-2 could be of potential therapeutic benefit, based on the comparative in vivo reports on similar viruses. 

NPs provide stabilization and the discharge of active ingredients of vaccines in the body; thus, NPs can also operate as carriers. Among certain types of respiratory diseases, NPs have been used in vaccines to treat respiratory syncytial virus (e.g., polyanhydrides), H1N1 influenza and human parainfluenza virus type 3 [214]. Further studies of the antiviral activity of AgNPs may reveal further potential in the treatment of diseases caused by a variety of viruses, especially COVID-19.

## 5. Applications of AgNPs

In the last few decades, it has been shown that biosynthesized AgNPs have potential uses in agriculture, health, food and industry because of their unique characteristics. A joint venture of the Food and Agriculture Organization (FAO) and WHO in 2009 presented the applications of nanotechnology in agriculture and food, with the addition of across-the-board fields such as food packaging, nanostructured ingredients, nanocoatings, nanosized biofortification and nanofiltration [215]. 

### 5.1. AgNPs in Agriculture

In adopting the 2030 plan for sustainable development, the United Nations dedicated itself to abolishing poverty and hunger and making agriculture sustainable. The global population is expected to grow to 9.8 billion by 2050; feeding such a huge population will necessitate at least a 50% rise in the production of agricultural products from 2012 levels by the mid-century [216]. Diverse nano-enabled measures are planned to progress crop production and facilitate the necessary increase in production for food, feed and fuel while practicing sustainable agriculture. NPs are considered “magic-bullets” to increase the production of agriculture as they contain nutrients, valuable genes and organic compounds that can be tailored towards precise plant structures or areas. Consequently, NPs represent elegant nanodelivery systems for agriculture administration, especially for crop nutrition. Following the poor outcomes of the “First Green Revolution” during the 1970s, there is an urgent need for a “Second Green Revolution” with an eco-friendly and more sustainable strategy. In agriculture, huge economic losses are caused by assorted plant diseases. After the first green revolution, the concept of biopesticides emerged to combat the arbitrary use and ill effects of chemical pesticides [217]. AgNPs are principally used for plant disease management because of their unique antimicrobial properties [218]. Many researchers have proposed the exploitation of the antimicrobial activities of AgNPs that are hostile towards a variety of plant pathogens. 

Research has been focused on the direct applications of AgNPs in agriculture, such as root elongation, seed germination, nanofertilizers, nanopesticides and plant modification (cytotoxicity or cellular oxidative stress) in the presence of metal NPs [117,219] and indirect usage based on the antimicrobial activities of NPs [220]. To boost crop yields, the use of chemical fertilizers is key, but their effectiveness is restricted by the occurrence of leaching or volatilization, which contaminates the environment and raises the cost of production. Therefore, nanofertilizers are gaining significant attention in sustainable agriculture as a way to extend the use of slow-release fertilizers, minimize the loss of mobile nutrients and facilitate access to poorly available nutrients [221]. In agriculture, in order to collect real-time statistics on crop growth, nutrients and water availability, nanosensing devices have been developed. Moreover, to reduce the use of chemicals on seeds and crops, carbon nanotubes (CNTs) and mesoporous silica nanoparticles have been applied [8]. 

The formulation of traditional pesticides with polymers or metal nanoparticles is an emerging area within the pesticide industry. The advantage of nanoencapsulation is the slow and reduced release of active ingredients by the use of nanocapsules, which minimizes surplus overflow of redundant pesticides and instead uses low doses over a prolonged time period [222]. An additional benefit of nanocarriers in plant protection is their site-targeted delivery and the stability of active ingredients [223]. In agriculture, AgNPs can be used as an ecologically sound strategy to replace pesticides and synthetic additives, as small concentrations of AgNPs are effective against pathogens, without toxicity to humans. Scientists have reported the broad-spectrum phytopathogenicity of AgNPs against various phytopathogens such as *Scalerotinia sclerotiorum*, *Fusarium culmorum, Trichoderma sp., Botrytis cinerea, Colletotrichum gloeosporioides, Rhizoctonia solani, Biploaris sorokinniana, Sphaerotheca pannasa, Phythium ultimum, Phoma* and *Megnaporthe grisea* [224,225,226].

A study has reported the application of phytosynthesized (kaffir lime leaf extract) AgNPs for boosting the germination and starch metabolism of matured rice seeds via nanopriming technology. The formulation of elevated soluble sugar content for sustaining the seedling development was made possible as the nanopriming stimulated α-amylase activity [227]. In another study, researchers explored the role of AgNPs in the development of seed germination and its effect on the plumule and radical length of *Pennisetum glaucum* [228]. The study revealed the importance of biosynthesized AgNPs, which can play a significant role in controlling *Acidovorax oryzae* strain RS-2. AgNPs downregulate the expression of type VI secretion system allied genes, which affects the virulence of bacteria [229]. Global efforts are underway to reduce the use of dangerous substances, especially chemical pesticides, in crop production. The biological means of soil phytopathogens has been confirmed to be a suitable substitute for the use of chemical pesticides. Past experience suggests that the use of fungal strains is a reliable and sustainable approach for the synthesis of AgNPs. In a study, *Trichoderma viride* biosynthesized AgNPs acted as an appropriate and successful biocontrol agent against rice pathogenic fungi such as *Rhizoctonia solani* and *Fusarium moniliforme* and offered better crop and disease control [230].

Currently, improving the yield is the primary goal for plant breeders in order to meet the growing harvest demand. To accelerate crop development, advanced biotechnological techniques such as next-generation sequencing (NGS) and genome-assisted breeding (GAB) are being used for the detection and assessment of genetic variability. Alternation of gene expression via DNA and RNA isolation in plants can be used for the development of biotic and abiotic stress-resilient crops [231]. However, poor cell membrane penetration and nucleus targeting are major challenges for the progression of plant-targeted delivery systems. The meeting of biotechnology with nanotechnology could help to overcome these challenges. Nowadays, NPs are being used as an efficient gene transformation vehicle. SiO_2_ NPs have been used to transport DNA fragments into corn and tobacco plants, without any harmful side effects [232]. The higher DNA delivery efficiency into plants (*Nicotiana tabacum*, *Oryza sativa* and *Leucaena leucocephala*) by AuNPs embedded in sharp carbonaceous carriers was established [233]. Thus, through desirable modification of plants, the future demand for plant-derived products can be met using nanobiotechnology applications. Nanocoating-based materials can play a significant role in agro-machinery applications by improving their resistance to aerial oxidation-based deterioration and UV rays. A future application could be in smart machines for specific weed management through the inclusion of nanobiosensors [231]. 

### 5.2. Food and AgNPs

Nanopotentials have been found to improve food characteristics, safety and production efficiency in food industries throughout the world. The antimicrobial efficacy of AgNPs has been widely explored in the food industries. Green synthesized AgNPs are considered safer or less toxic for potential applications in food items [234]. The use of several Ag-containing food packaging materials has been approved by the USFDA in order to preserve the shelf life of food items and remain in direct contact with food items [235]. The safety and freshness of fruits and vegetables is one of the major problems faced in the agri-food sector. The inability of natural wax coatings to prevent water loss and reduce the respiration rate causes protein and weight losses during the long-term storage of fruits and vegetables. The shelf life of different food items can be significantly improved by the usage of various protective nanocoatings and appropriate packaging materials [1]. Aseptic food packaging materials can be developed by the use AgNPs, as AgNPs exhibit a broad array of antibacterial actions. Researchers increased the shelf life of fruits and vegetables by incorporating mycosynthesized (*Trichoderma viride*) AgNPs into sodium alginate, which prevented the protein and weight loss and also avoided microbial spoilage [236]. Similarly, another study reported the improved shelf life of chikku and grapes with the thin film of *A. niger* biosynthesized AgNPs in combination with sodium alginate [237]. 

### 5.3. Biomedical Applications

The unique physicochemical and antimicrobial properties of AgNPs have found a wide range of biomedical applications, such as in pharmacology, drug delivery, diagnostics, anticancer, etc. Garcia-Contreras et al. (2011) reported the exploitation of AgNPs in dental practices, such as in endodontic retrofill cement, dental implants and restoring material [238]. AgNPs’ unique toxicity profile can enable the targeting of specific vulnerabilities in cancer due to their negligible systemic toxicity. Several studies reported the beneficial role of AgNPs in cancer therapy. The toxic effects of biosynthesized AgNPs against carcinoma cells compared to non-cancer cells were studied [239]. Another study showed the significant inhibition of cancer cell lines (B16F10, A549, MCF7 and HNGC2F) after treatment with AgNPs synthesized with *Butea monosperma* leaf extract [240]. The toxicity profiling of AgNPs on ovarian cancer stem cells was investigated and outcomes showed that A2780 and ALDH^+^/CD133^+^ colonies were reduced significantly [241]. 

Inflammation is the reaction of the immune system to infections and injury to cells, which helps in tissue organization and the elimination of harmful factors [242]. An inflammatory disorder can develop due to the disruption of this complex process [243]. Anti-inflammatory compounds such as cytokinins and interleukins are produced by the primary immune organs in response to inflammatory disorders [244,245]. Biologically synthesized AgNPs possess these anti-inflammatory activities because the alkaloid or flavanoid contents function as capping agents and provide supplementary pharmacological properties. Nanosilver in higher doses has been used to facilitate the strong production of Th1 cells by the secretion of IL-2 and INF-γ, which play a crucial role in cellular immunity [243]. A research study was carried to evaluate the anti-inflammatory action of AgNPs synthesized by *Leucas aspera* plant. Ethanol and AgNPs of *L. aspera* demonstrated anti-inflammatory action against carrageenan-induced paw-edema in rats and indomethacin was used as a standard drug. Excellent anti-inflammatory activities were reported using *L. aspera* biosynthesized AgNPs [246]. Kim et al. (2007) used Muller Hinton agar plates to study the antimicrobial activities of AgNPs against *E. coli*, *Staphylococcus aureus* and yeast, showing that they are suitable for various medical devices and antimicrobial control systems [247]. 

### 5.4. Environment and AgNPs

The biosynthesized AgNPs have emerged as novel tools for the development of nanobiosensors to target environmental pollutants. The detection of residual pesticides in environmental samples has become possible via the surface modification of nanoparticles with an appropriate agent. For the detection of pesticides, nanotechnology-based colorimetric methods offer several advantages, such as accuracy, no need for specialized equipment, simplicity, less time consumption, etc. The specificity and sensitivity of nanoparticle-based colorimetric detection methodology is based on the chemical and molecular connections among the surface-modified nanoparticle and target pesticides [248]. The high extinction coefficient and strong surface plasmon resonance of silver (Ag) have attracted interest for the recognition of pesticide residues in the environment [249,250]. Xiong and Li described a colorimetric probe for the quantitative detection of residual pesticides by means of calixarene-modified AgNPs. The preparation of extremely firm calixarene-modified silver nanoparticles (pSC_4_-Ag NPs) was achieved by a one-pot synthesis scheme and they were then characterized. pSC_4_-Ag NPs have been proposed as a new sensor for the colorimetric recognition of residual pesticides in water, with an optunal down to a concentration of 10^−7^ M [251]. Similarly, Menon et al. (2013) reported a straightforward and extremely sensitive method for the detection of dimethoate pesticides in industrial wastewater via p-sulphonate calyx resorcinarene customized silver nanoparticles (pSC_4_R-Ag NPs) [252].

## 6. Cytotoxicity and Safety Issues of AgNPs

The exploitation of AgNPs is in the infancy stage and the overall evaluation of their health hazards is so far not promising, although a number of products are already on the market. In the immediate future, we expect that the use of AgNPs in human health will increase significantly because of their utility, principally in relation to pandemic control. The lethal outcomes of AgNPs in organisms depends on several factors, such as the route of exposure (penetration, concentration and duration), factors associated with vulnerable organisms and factors associated with AgNPs’ intrinsic toxicity, bioavailability and accumulation in organisms [253]. Three possible methods of consuming AgNPs are: inhalation, parenteral/dermal and oral. The cytotoxic effects of AgNPs depend on their size, tissue allocation, infiltration competence and cellular absorption [254]. 

Once in the human body, large AgNPs can be exhaled, while the smaller AgNPs can be deposited in the lungs and can reach different organs via the bloodstream. According to histopathological data studies, no considerable changes were found in the lungs, liver, nasal cavities or other organs regarding AgNPs with average sizes of 15–30 nm and in concentrations of 0.5–381 µg/m^3^ [255,256,257,258]. However, brain injuries have been reported at high concentrations of AgNPs above 2.9 mg/m^3^ [259]. 

The adhesiveness of AgNPs in biological tissues also depends on the presence of an electric charge on the NPs’ surfaces [260]. Negatively charged NPs show quite low DNA encapsulation in comparison to positively charged NPs, which attach to DNA plasmids via electrostatic contacts and increase their stability. AgNPs also form electrostatic contacts with certain blood proteins, ions and other components [261]. 

The level of AgNPs’ cytotoxicity in dermal injections rises with a concentration of 0.1–1000 mg/kg and size of 20–100 nm, causing cerebral [262], lung [263], renal [264] and liver lesions [265]. 

Oral ingestion involves an intermediate level of toxicity, where the NP dosage ranges (0.5–500 mg/L) were decisive rather than their size. Various research studies showed the following effects of NPs doses: weight loss at doses of 10 mg/kg [266], liver disorders at doses >300 mg/kg [267] and oxidative stress generated at >1000 mg/kg doses of NPs [268]. In oral ingestion, most of the applied doses of NPs are ultimately expelled in the feces; hence, accumulation is least comparable to dermal injection [269]. Vazques-Munoz et al. (2015) investigated AgNPs’ toxicity in a wide range of biological entities ranging from virus to human cell lines. This study revealed that different biological entities were inhibited within the same order of magnitude (10 µg/mL) of AgNPs. This was because of the interactions of AgNPs with fundamental components of cells and viruses alike [270]. 

The transfer of in vivo toxicological knowledge regarding dosages of AgNPs from rats to humans is of immense significance, because not many studies have been performed concerning human participants. Using AgNP exposure concentrations relative to deposited mass per alveolar surface area, correlation is made via multipath particle dosimetry modeling. The alveolar surface area of rats is 0.409 m^2^, while in the case of humans, it is 62.7 m^2^ [271]. Certain studies suggest that experience time is not the most important aspect to determine AgNPs’ cytotoxicity for humans, because, in certain cases, no blood or hematological changes were observed even when exposure exceeded 5 years with concentrations of 0.35 and 1.35 mg/m^3^ [272]. A report suggested that a jewelry manufacturer works at a much lower concentration of AgNPs (0.2–2.8 mg/L) in comparison to an individual who recovers Ag in close proximity to soluble compounds, with a concentration of 1.3–20 mg/L. Therefore, an extended experience times does not influence the health of workers; rather, exposure depends on the activity. At moderate exposure, blood AgNP levels should be in the range of 0.1–23 mg/L, whereas sporadic exposure should be around ≤0.1 mg/L [272,273]. Nevertheless, research studies on the cytotoxicity of NPs need to be expanded, particularly focusing on their effects in the lungs, since NP exposure occurs primarily through inhalation. 

## 7. Conclusions and Future Prospects

AgNPs have long been of interest to researchers due to their customizable properties. The enhanced properties of green synthesized AgNPs can be exploited for use in almost all areas of science and everyday life, including preventing epidemics and treating contagious diseases. This article comprehensively reviewed the updated progress in AgNP biosynthesis approaches, their antimicrobial activities and their applications in various fields. The underlying concepts behind biosynthesized AgNPs, their antimicrobial activities and their cytotoxicity have been studied carefully with regard to the mechanisms, types and factors controlling the nanosynthesis process and their actions. Better-quality nanomaterials are formulated via intracellular biosynthesis approaches. Nonetheless, the downstream processing of intracellular nanoparticles is highly expensive and difficult in comparison to extracellular methods. Further studies are required to elucidate the detailed mechanisms of AgNP production to achieve pure nanoparticles, as well as to determine their stability and the functions of the metabolites produced by the microorganisms. This review also highlights the applications of AgNPs in terms of SARS-CoV-2 and suggests that future studies could play a prominent role in driving the myriad microorganisms and plants explored till date to achieve their on-field applications. The inhibitory effect of AgNPs on SARS-CoV-2 could lead to a novel scientific approach for preventing infections at an early stage. There is also emerging evidence of the ability of AgNP coatings to reduce their cytotoxicity and increase their specificity. Therefore, more research is needed on the effective doses and probable toxic effects of AgNPs to create a safe environment for humans against extremely dangerous diseases such as SARS-CoV-2. Lastly, a better understanding of the cytotoxicity and safety issues associated with AgNPs towards humans and the environment could further the limits of this technology and expand their horizon beyond laboratory use. 

## Figures and Tables

**Table 1:** Bacterial strains used for the biosynthesis of AgNPs, their characterization and activities.

Sr. No.	Organism Taken for AgNPs	Size	Shape	Characterization	Synthesis Conditions	Activity Studied	Reference
1.	*Escherichia coli*, *Exiguobacterium aurantiacumm*, *Brevundimonas diminuta*	5–50	Spherical	UV, TEM, XRD, FTIR, SEM	At pH 9, incubation temp. 37 °C, for 24 h and 72 h in dark	Antibacterial Gram-positive *Bacillus subtilis*, *Staphylococcus aureus*, *Bacillus cereus* Gram-negative *Staphylococcus*, *Pseudomonas aeruginosa*, *Klebsiella pneumonia*, *Escherichia coli*, *Salmonella typhi*, *Enterobacter vermicularris*	[18]
2.	Cyanobacterium, *Chroococcus* *minutus*			UV, SEM, FTIR, XRD, SEM-EDX	Incubated for 24–56 h at 40 °C	Antibacterial *Escherichia coli*, *Staphylococcus aureus*, *Pseudomonas aeruginosa*	[21]
3.	*Lysinibacillus* *xylanilyticus* strain MAHAQ-40	8–30	Spherical	UV, FTIR, XRD, DSL, FE-TEM	Incubated for 48 h at 30 °C	Antibacterial *Salmonella typhimulium*, *Vibrio parahaemolyticus*	[19]
4.	*Sphingobium sp. * MAH-11	7–22	Spherical	SAED, XRD, FTIR	Incubated for 24 h at 30 °C	Antibacterial *Staphylococcus aureus*, *Escherichia coli*, *Pseudomonas aeruginosa*	[22]
5.	*Bacillus pumilus*, *Bacillus* *paralicheniformis*, *Sphigomonaspaucimobilis*	4–20	Spherical, Oval	XRD, TEM, FTIR		Cytotoxicity *Vigna radiata*	[23]
6.	*Streptomyces* *coelicoflavus* KS-3	2.33–91.3	Spherical, truncated, triangular, quadrangular and hexagonal	XRD, TEM, FTIR, DLS, EDX	At pH 7, incubated for 72 h at 32 °C in dark	Cytotoxicity Carcinoma cells (HTB-182), Adeno-carcinoma cells (A549)	[20]
7.	*Bacillus subtilis* (SDUM301120)	2–26	Spherical	HRTEM, UV, XRD EDX, FTIR	At pH 9, incubated for 12 h	Antibacterial *Escherichia coli* ATCC 25922, *Staphylococcus aureus* ATCC29213, *Vibrio parahaemolyticus* ATCC 17802, *Acinetobacter baumanni* ATCC 19606	[24]
8.	*Cytobacillus* *fimus*	14.23	Spherical	UV, FTIR, XRD, SEM, DLS	Incubated for 24 h at 37 °C	Antibacterial *Escherichia coli*, *Staphylococcus aureus* Antifungal *Magnaporthe grisea*	[25]
9.	*Lysinibacillus* *sphaerius*	14–21	Spherical, hexagonal, cuboidal, rod-shaped, irregular	UV, TEM, DLS, FTIR	Incubated at room temp. for 48 h	Antibacterial Gram-negative *E. coli* ATCC 25955, *Pseudo aeruginosa* ATCC 10145, *Klebsiella pneumonia*, *Proteus vulgaris*, *Salmonella typhimurium*, *Enterobacter aerogennes*, *Shigella dysenteriae* Gram-positive *Bacillus subtilis* ATCC 6633, *Staphylococcus aureus* NRRL Antifungal Yeast, filamentous fungi Virucidal *Rotavirus* Cytotoxicity Epithelial cell MA 104	[26]
10.	*Bacillus brevis* (NCIM 2533)	41–68	Spherical	UV, FTIR, TLC, SEM, AFM	Incubate for 2 h at room temp.	Antibacterial *Salmonella typhai*, *Staphylococcus aureus*	[27]
11.	*Bacillus pumilus*, *B. persicus*, *B. licheniformis*	77–92	Spherical, triangular, hexagonal	UV–Vis, FTIR, TEM, EDX, DLS		Human pathogenic bacteria Bean yellow mosaic virus	[28]

**Table 2:** Algal species used for the biosynthesis of AgNPs, their characterization and activities.

Sr. No.	Organism Taken for AgNPs	Size (nm)	Shape	Characterization	Synthesis Conditions	Activity Studied	References
1.	*Pyropiayozoensis*	20–22	Spherical	FTIR, XRD, SEM, TEM	Incubated at 35 °C in dark for 15–20 min	Antibacterial *Pseudomonas aeruginosa*, *Staphylococcus aureus*	[39]
2.	*Polysiphonia*	25	Spherical	FTIR, SEM, TEM, EDX	Stirring at room temp. for 2 h	Anticancer MCG-7 cell line	[40]
3.	*Spirulina platensis*	30–50	Spherical	FTIR, UV, SEM, TEM	At pH range 4.7–5.0, incubated for 10 min at 60 °C	Antibacterial *Escherichia coli*, MTCC-9721, *Proteus vulgaris*, MTCC-7299, *Klebsiella pneumoniae*, MTCC-9751, *Staphylococcus aureus*, MTCC-9542, *S. epidermidis*, MTCC-2639, *Bacillus cereus*, MTCC-9017	[41]
4.	*Chlorella valgaris*	55.06–61.89	Spherical	FTIR, XRD, FESEM, UV, DLS	Incubated at room temp. for 24 h	Photocatalytic dye degradation	[42]
5.	*Spyridia fusiformis*	5–50	Spherical, triangular, pseudo-spherical, rectangular	FTIR, TEM, XRD, HR-TEM	Incubated at room temp.	Antibacterial *Escherichia coli* (ATCC 10798), *Klebsiella pneumaniae* (ATCC 31488), *Staphylococcus aureus* (ATCC 10832D-5), *Pseudomonas aeruginosa* (ATCC 207)	[43]
6.	*Noctiluca valgaris*	4.5	Spherical	DSL, SEM, EDS, UV, HRTEM		Anticancer MDA-MB-231 Antibacterial *Escherichia coli* ATCC25922, *Staphylococcus aureus* ATCC29213	[44]
7.	*Spirulina platensis*	29	Spherical and dispersed	UV, SEM, TEM, DSL, XRD, FTIR	Under sunlight for 10–20 min at pH 7, incubated at room temp.	Anti-biofilm *Pseudomonas aeruginosa* PA14	[45]

**Table 3:** Fungal species used for the biosynthesis of AgNPs, their characterization and activities.

Sr. No.	Organism Taken for AgNPs	Size (nm)	Shape	Characterization	Synthesis Conditions	Activity Studied	Reference
1.	*Trichoderma* *spp.*		Round	UV, TEM, FTIR	pH 5–7 Incubated for 3–9 days at 32 °C	Antibacterial Gram-positive *Staohylococcus aureus* ATCC 6538, *Enterococcus faecalis* ATCC 29212 Gram-negative *E. coli* ATCC 8939, *Pseudomonas aeruginosa* ATCC25853	[56]
2.	*Penicillium* *aculeatum* Su1	4–55	Spherical	UV, TEM, DLS, EDX, XRD, FTIR	Incubated for 72 h in dark at 32 °C	Enzyme activity Nitrate reductase Protein identification	[57]
3.	*Trichoderma* *longibrachiatum*	10	Spherical	UV, TEM, FTIR, DLS	Incubated for 48 h in dark at 22–33 °C	Antifungal *Fusarium verticillioides*, *Fusarium moniliforme*, *Penicillum brevicompactum*, *Heminthosporium oryzae*, *Pyricularia grisea*	[50]
4.	*Fusarium* *scirpi*	2–20	Quasi- spherical	UV, XRD, STEM, HRTEM, EDX	Incubated for 72 h at 28 °C	Antimicrobial Uropathogenic *E. coli*	[58]
5.	*Punica* *gramatum*	5–45	Spherical	UV, FTIR, XPS, XRD, TEM	Incubated for 2 days at 90 °C	DPPH, DNA cleavage, Antibacterial Gram-negative *L. pneumophila*, *P. aeruginosa*, *E. coli* Gram-positive *E. hirae*, *B. cereus*, *S. aureus*	[55]
6.	*Pichia kudriavzevii HA-NY2* *Saccharomyces* *uvarum* HA-NY3	12.4–30	Cubic, spherical	UV, TEM, FTIR, DLS	Incubated for 72 h in dark at 30 °C	Antibacterial Gram-positive *Bacillus subtilis* ATCC 6633, *Staphylococcus aureus* ATCC 29213 Gram-negative *Pseudomonas aeruginosa* ATCC 27953, *Candida tropicalis* ATCC 750, *Fusarium oxysporium* NRC21 Anti-inflammatory *Paw edema*	[54]
7.	*Aspergillus niger*	10.31	Spherical	FTIR, TEM, UV	Incubated for 72 h in dark at 28 °C	Anti-amoebic *Allovahlkampfia spelaea*	[59]
8.	*Piriformospora* *indica*	6–15	Spherical	UV, SEM, EDX, TEM, FTIR, XRD	At pH 6, incubated for 72 h at 28 °C	DPPH Anticancer Human breast Adenocarcinoma (MCF-7), Human cervical carcinoma (HeLa), Human liver hepatocellular carcinoma (HepG2), Embryonic kidney cell (HEK-2930) Antiproliferative MCF-7, HeLa, HepG2	[60]
9.	*Trichoderma*	5–50	Spherical, oval	SEM, EDS, TEM, XRD, FTIR	At pH 7, incubated for 1 h at 25 °C	Antifungal *Sclerotinia sclerotiorum*	[61]
10.	*Cinnamomum* *zeylanium*	76	Spherical, oval	SEM, EDS, TEM, XRD, FTIR, EDX	Incubated for 24 h at room temp.	Antibacterial *Staphylococcus aureus*, *E. coli*, *Pseudomonas aeruginosa*	[53]

**Table 4:** Plant species used for the biosynthesis of AgNPs, their characterization and activities.

Sr. No.	Plants	Part	Size (nm)	Shape	Characterization	Reducing Agent	Synthesis Conditions	Activities Studied	References
1	*Ruta graveolens*	Leaves	40–45 nm	Spherical	FTIR, SEM	Alcohol, phenol, primary amine, sec. amine, azide, carbamide, allene, ketenimine, alkane, alkene, aldehyde, ester, amine, halo compounds	Incubated at room temp. for 24 h	Anticancer Antibacterial *Escherichia coli*, *Proteus mirabilis*, *Shigella flexneri*, *Staphylococcus aureus* Insecticidal *Culex pipiens*	[66]
2	*Phoenix dactylifera*, *Ferula* *asafetida*, *Acacia nilotica*	Fruit	67.8–155.7 nm	Spherical	FT-IR FE-SEM, TEM, Zeta potential	Alcohol, phenol or glycoside, amide, aromatic nitrile	Incubated at room temp. for 48 h	Antibacterial *Escherichia coli*, *Staphylococcus aureus*, *Pseudomonas aeruginosa* Anticancer	[69]
3	*Cymbopogon citrates* (lemon grass)	Leaves	50–80 nm	Spherical	UV–Vis, XRD, FTIR, AFM, SEM, TEM	Amine, phenol, alkane, alkyl	Incubated at room temp.	Antidiabetic	[70]
4	*Aaronsohnia factorovskyi*	Stem, leaves, flower	104–140 nm	Spherical	UV–Vis, FT-IR, FE-SEM, GC–MS	Carboxylic acid, alkyne, thiocynante, aronatic compound, alkene, isothiocynate	Incubated in sunlight for 30 min	Antibacterial *Staphylococcus aureus*, *Bacillus subtilis*, *Pseudomonas aeruginosa, Escherichia coli* Antifungal *Fusarium oxysporum*, *Fusarium solani*, *Helminthosporum rostratum, Alternaria alternate*	[71]
5	*Eucalyptus camaldulensis*	Leaves	16–28 nm	Spherical	UV–Vis, SEM, FTIR, XRD, EDX, DLS, Zeta	Alcohol, phenol, flavonoid, flavones, catechin, ester, ether, alkane, carboxylic acid, primary amine, aldehyde	Incubated at 25 °C in dark for 24 h	Antioxidant	[72]
6	*Terminalia arjuna*	Leaves	10–50 nm	Spherical	UV–Vis, FTIR, TEM, FE-SEM, XRD	Halo compounds, amine, alkyne, alcohol, phenol	Incubated at 40–45 °C	Catalytic degradation of organic dyes methyl orange, methylene blue, Congo red and 4-Nitrophenol	[73]
7	*Ehretialaevis*-Roxb.	Leaves	25–30 nm	Spherical	UV–Vis, FTIR, TEM, XRD, EDX, Zeta	Alcohol or phenol, alkenes, primary amines, alkanes, alkyl halides	Incubated at 90 °C for 1 h	Antimicrobial *Bacillus subtilis*, *Escherichia coli*, *Enterococcus faecalis*, *Pseudomonas arginosa* Larvicidal *Culex quinquefasciatus* Anticancer	[74]
8	*Capparis zeylania* L.	Leaves	23 nm	Spherical	UV–Vis, FTIR, XRD, SEM, TEM	Alkynes, phosphine group, aliphatic ester, amine, nh group, carbonyl group, hydroxyl group	Incubated in dark conditions at 37 °C	Antibacterial *Enterococcus faecalis*, *Staphylococcus epidermidis*, *Salmonella paratyphi*, *Shigella dysenteriae* Antifungal *Candida albicans*, *Aspergillus niger* Antiproliferative	[75]
9	*Tribulus terrestris* L.	Shoot	~25 nm	Spherical	UV–Vis, TEM, DLS, XRD		Kept in dark at room temp.	Antibacterial *Bacillus subtilis*, *Escherichia coli*, *Pseudomonas aeruginosa*, *Staphylococcus aureus* Photocatalytic activity Cytotoxicity activity	[76]
10	*Cyperus pangorei*	Leaves	32–60 nm	Spherical	UV–Vis, FTIR, XRD, TEM, EDXXPS	Carboxylic acid, phenol group, cyclohexane ring	Kept in an oven at 100 °C for 1 h	Photocatalytic activity of Ag NPs against dye Rhodamine B	[77]
11	*Onosmasericeum* Wild.	Root	10–50 nm	Spherical	UV–Vis, FT-IR, FESEM, EDAX, TEM, XRD	Alcohol or phenol	pH 8, temp. 25 °C	Antibacterial *Acinetobacter baumannii*, *Bacillus subtilis*, *Escherichia coli*, *Staphylococcus aureus*, *Aeromonas hydrophila* Cytotoxicity MCF-7 breast cancer cell line Catalytic effect 2-nitrobenzenamine	[78]
12	*Abutilon indicum*	Leaves	50–100 nm	Spherical	UV–Vis, FTIR, SEM	Amines, alcohol, ketones, aldehyde, etc.	Incubated at room temp.	Antibacterial *Escherichia coli*, *Streptococcus aurous*	[79]
13	*Decaschistiacrotonifolia*	Leaves	12–18 nm	Spherical	UV–Vis, FTIR, TEM, XRD	Alcohol, carboxylic acid, ester, ether, phenols, alkanes, amides, alkaloid, etc.	Incubated at room temp.	Antimicrobial *Escherichia coli*, *Staphylococcus aureus, Klebsiella pneumonia*, *Bacillus subtilis* Antioxidant Photocatalytic activity of dyes Cotton blue Congo red 4-nitrophenol	[67]
14	*Azadirachta indica*, *Citrullus colocynthis*	Leaves Fruit	17 nm 26 nm	Spherical	UV–Vis, FTIR, SEM, EDX, XRD	Polyphenols, aromatic terpenoid, flavonoids, alkene, ether, amines, aldehydes, ketones, carboxylic acid	70 °C temp.	Larvicidal *Aedes aegypti*	[80]
15	*Litchi chinensis*	Leaves	5–15 nm	Spherical	UV–Vis, FTIR, TEM	Phenolic compound, alcohol, etc.	95 °C temp.	Bactericidal and sporicidal *Bacillus subtillus*	[6]
16	*Asphodelus tenufolius*	Shoot, Seed	58.2 nm 51.6 nm	Spherical and polydispersed	UV–Vis, FTIR, SEM	Alkanes, alkyne, alkene, carboxylic acid, alcohol/ phenols	pH 5.5, temp. 30 °C		[81]
17	*Jasmine flower*	Flower	40 nm		UV–Vis, FTIR, SEM, TEM	Phenolic compounds, alcohol, phenol, alkyl, etc.	Incubated for 2 h at 110 °C	Antimicrobial *Escherichia coli*, *Staphylococcus aureus* Photocatalytic degradation of methylene blue dye	[82]
18	*Pisum sativum* L.	Seed	10–25 nm	Spherical	UV–Vis, FTIR, XRD, SEM	Phenol, alkynes, amines, alkyl halides, etc.	24 h incubation	Antioxidant Antidiabetic Cytotoxicity Antibacterial *Escherichia coli*, *Enterococcus faecium*, *Streptococcus typhimurium* and *Streptococcus entrica*	[83]
19	*Eryngium caucasicumTrautv*	Leaves	10–20 nm	Spherical	UV–Vis, FTIR, XRD, TEM, SEM	Phenols, amines, alcohol, carboxylic acid, ester, ether, terpenoid, flavonoids, tannins	Incubated at 80 °C for 8 h	Antibacterial *Escherichia coli*, *Staphylococcus aureus*	[84]
20	*Dregavolubilis*	Flower	8.59–19.18 nm	Spherical	UV–Vis, FTIR, FESEM, EDX, HRTEM	Polyphenol, phenolic acid, polysaccharides, flavones, amide	Incubated at room temp.	Antioxidant Antidiabetic Antibacterial *Bacillus subtilis*, *Escherichia coli*, *Pseudomonas aeruginosa*, *Staphylococcus aureus*	[85]
21	*Carissa carandas L.*	*Leaves*	30–35 nm		UV–Vis, FTIR, XRD	Alkenes, methoxy group, alkynes	Incubated at temp. 25 and 60 °C	Antioxidant Antibacterial *Salmonella typhimurium*, *Enterobacter faecalis*, *Shigella flexneri*, *Citrobacter spp.* *Gonococci spp.*	[68]
22	*Artemisia vulgaris*	Leaves	25 nm	Spherical	UV–Vis, FTIR, SEM, EDX, TEM, AFM	Phenolic group, phenols, aromatic amines, carbonyl groups	Incubated at room temp. for 2 h	Antioxidant Anticancer Antimicrobial *Escherichia coli*, *Pseudomonas aeruginosa*, *Staphylococcus aureus*, *Klebsiella pneumoniae*, *Haemophillus influenza*	[86]
23	*Aervalanata*	Flower	90 nm		UV–Vis, SEM, AFM, FTIR, TEM, XRD	Alkenes, secondary amines, carboxylic acid ether, ester alcohol		Anticancer Antibacterial *Bacillus subtilis*, *Escherichia coli*, *Klebsiella planticola*, *Streptococcus faecalis* Photocatalytic activity Methylene blue	[87]
24	*Moringa oleifera*	Seed	4.0 nm	Spherical	UV–Vis, SEM, TEMFTIR, XRD	Polyphenolic or flavonoid compounds, alkanes, alkenes, primary alcohol	pH 11.0, temp. 60 °C	Antimicrobial *Escherichia coli*, *Pseudomonas aeruginosa*, *Salmonella enterica typhimurium* Photocatalytic activity Methylene blue, orange red, 4-nitrophenol	[88]
25	*Euphorbia sanguine*	Leaves	20–28.8 nm	Spherical	UV–Vis, SEM, TEM, FTIR	Amines, hydroxyl group	Incubated at room temp.	Photocatalytic activity Congo red dye Melanogenesis inhibition activity	[89]
26	*Annona reticulata*	Leaves	7.67–8.34 nm	spherical	UV–Vis, FTRI, TEMXRD	Chloride group, anhydride group, methyl group, carbonyl group, alkane, amide	Incubated for 2 h in dark at room temp.	Larvicidal *Aedes aegypti* Antibacterial *Escherichia coli*, *Pseudomonas aeruginosa*, *Staphylococcus aureus*, *Bacillus cereus* Antifungal *Candida albicans*	[90]
27	*Allium ampeloprasum*	Aerial part	2.3–27 nm	Spherical	UV–Vis, FTIR, XRD, TEM	Alcohol, phenolic compound, methyl, methylene, methoxy group, carboxylic acid, ester, ether, aliphatic amine	Temp. 35–37 °C	Anticancer Antibacterial *Escherichia coli*, *Pseudomonas aeruginosa*, *Staphylococcus aureus* Antioxidant	[91]
28	*Chlorophytum borivilianum*	Root tuber	2.4–19.4 nm	Spherical	UV–Vis, FTIR, XRD, TEM	Hydroxyl group, aldehyde group, amide, aromatic ether	pH 4.6, at room temp.	Phytotoxicity during seedling growth of *Peltophorumpterocarpum*	[92]
29	*Rosa Santana* (Rose)	Petal	6.52–25.24 nm	Spherical	UV–Vis, FTIR, XRD, TEM	Hydroxyl, alkyl, alkyne, halogenated compound	Incubated at 90 °C for 25 min	Antibacterial *Escherichia coli*, *Staphylococcus aureus* Cytotoxicity against mouse fibroblast cell line by XTT assay	[93]
30	*Citrus medica*, *Tagetes lemmonii*, *Tarenna asiatica*	Leaves	40–220 nm, 30–120 nm, 60–350 nm	Spherical, cuboid	UV–Vis, FTIR, SEM, XRD	Aromatic amines, alcohols, carboxylic acids, esters, ether		Larvicidal *Aedes aegypti*	[94]
31	*Diospyros lotus*	Leaves	10–25 nm	Spherical	UV–Vis, FTIR, TEM, XRD, SEM	Phenolic, aromatic alkenes, aliphatic hydrocarbon chain, amine		Phytochemical screening Antibacterial *Escherichia coli* Anticoagulant Catalytic activity Methylene blue	[95]
32	*Chlorophytum borivilianum* L. (Safed musli)	Callus	AVG. 52 nm		UV–Vis, FTIR, XRD, AFM	Amine, phenol, hydroxyl, alkyl, alkenes, alkynes	Incubated at room temp. for 5 h	Antibacterial *Escherichia coli*, *Pseudomonas aeruginosa*, *Staphylococcus aureus*, *Bacillus subtilis* Antifungal *Candida albicans* Cytotoxicity Colon cancer cell line	[96]
33	*Putranjivaroxburghi* Wall	Seed	13–69 nm	Spherical	UV–Vis, AEM, XRD, FTIR, TEM	Aliphatic primary amine, alkane, aldehydes, alkene, amines, sulfoxide, alcohol/phenols, phosphine, alkyl halide, alkyne, Staphylococcus	pH 8.5	Phytochemical analysis Anticancer Antibacterial *Escherichia coli*, *Staphylococcus aureus*, *Streptococcus pneumoniae, Enterococcus faecalis*	[97]
34	*Cataharanthus roseus*	Leaves			TEM		Incubated in dark at room temp. for 24 h	Anticancer	[98]
35	*Solanum turvum*	Fruits	27 nm	Spherical	UV–Vis, FTIR, DLS, SEM, EDS, TEM	Alcoholic and phenolic compound, alcohol and ether, polyphenols, amide, carbon chloride	Incubated at room temp. for 12 h, pH 6	Antibacterial *Xanthomonas axonopodispv. punicae*, *Ralstoniasolanacaerum* Phytotoxicity study *Vigna unguiculata*	[99]
36	*Acacia nilotica*	Stem	27–50 nm	Spherical	UV–Vis, FTIR, XRD, SEM, TEM, XPS, DLS, etc.	Phenol/carboxylic acid, secondary alcohol	Temp. 40–50 °C for 5 h	Antibacterial *Methicillin* resistance, *Staphylococcus aureus* Antifungal *Candida albicans* Reduction of pollutant 4-nitrophenol, 2-nitrophenol Degradation of dyes Congo red, methylene blue, methyl orange	[100]
37	Corn cobs	Xylan	Avg. 55.3 nm	Spherical, triangular, square, oval	UV–Vis, AFM, EDS, DLS, SEM, FTIR, RAMAN spectroscopy, flow cytometry	Monosaccharide, carboxylic group, beta-glycosidic bond, hydroxyl group	Incubated for 24 h in dark conditions	Anti-parasitic activity *Trypanosoma cruzi* Cytotoxicity	[101]
38	*Murrayakoenigii*	Leaves	Avg. 42 nm	Spherical	UV–Vis, FTIR, SEM	Hydroxyl group, ketones, aromatic compounds, quinone	Incubated at room temp. for 2 h	Antibacterial *Escherichia coli*, *Pseudomonas aeruginosa*, *Staphylococcus aureus*,*Klebsiella pneumoniae* Cytotoxicity In vitro In vivo	[102]
39	*Nigella sativa* (Black cumin)	Seed	Avg. 34 nm	Spherical	UV–Vis, XRD, FTIR, TEM, EDX, GC–MS	Hydroxyl, amide, alkenes, alcohol, aldehydes, ketones or carboxylic acid		Antidiabetic Anti-inflammatory Antioxidant Antibacterial *Escherichia coli*, *Pseudomonas aeruginosa*, *Staphylococcus aureus*, *Listeria monocytogenes*	[103]
40	*Catharanthus roseus*	Leaves	40–60 nm	Bunch form	FTIR, EDX, SEM			Antibacterial *Shigella dysenteriae*, *Klebsiella pneumoniae*, *Bacillus anthraces*, *Staphylococcus aureus*, *Pseudomonas aeruginosa*	[104]
41	*Allopylus cobs*	Leaves	2–100 nm	Crystalline	UV–Vis, XRD, FTIR, TEM, XPS, DLS	Methyl, amide, free amino, carboxylate		Antibacterial *Pseudomonas aeruginosa*, *Shigella flexneri*, *Staphylococcus aureus*, *Streptococcus pneumonia* Anti-biofilm	[105]

**Table 5:** Antiviral efficacy of AgNPs on different viruses, their characterization and activities.

Sr. No.	Virus	Family	Source of AgNPs	AgNPs Size	Composition	Synthesis Conditions	Mechanism of Action	Reference
1.	Coronavirus	Coronaviridae	Pure Ag Nanowire and colloid	10–20 nm	3–13 µg/mL	AgNPs procured from the Institute for Health and Consumer Protection (IHCP, a Joint Research Centre of European Commission located in Italy)	AgNPs decreased cell apoptosis through activation of p38/mitochondria/caspase-3 signaling in ST cells	[184]
2.	Malaria, Nile Virus, Zika	Flaviviridae	*Naregamiaalata*	5–35 nm	6–30 µg/mL	Incubated for 10 min at room temp.	AgNPs’ passage through the insect cuticle and into individual cells interferes with molting and other physiological processes	[185]
3.	SARS-CoV-2	Coronaviridae		200 nm coating	100–200 µL		Coating reduces the titers of SARS-CoV-2 to zero	[186]
4.	Herpes Simplex Virus and Human Parainfluenza Virus Type 3	Herpesviridae Paramyxoviridae	* Alternaria * species	46 nm	0.1–10 µg/mL	Fungus suspended in distilled water for 48 h	AgNPs control viral infectivity by blocking interaction of the virus with the cell	[187]
5.	Herpes Simplex Virus Human Parainfluenza Virus Type 3	Herpesviridae Paramyxoviridae	*Fusarium. oxysporum *	20 nm	0.1–10 µg/mL	Fungus suspended in distilled water for 48 h	AgNPs may block an early event before stable binding of the virus with the cell membrane, but it is likely that the nanoparticles interact directly with the viral envelope or its proteins and behave as virucidal agents	[187]
6.	Herpes Simplex Virus and Human Parainfluenza Virus Type 3	Herpesviridae Paramyxoviridae	* Curvularia * species	30 nm	0.1–10 µg/mL	Fungus suspended in distilled water for 48 h	Interference with replication at the post-entry phase	[187]
7.	Herpes Simplex Virus (HSV-I,II)	Herpesviridae	*Sargassum withtii*		0.5–5 µg/mL	Seaweed powder treated with 90 mL of 10 mM AgNO_3_ solution for 15 h under stirring conditions	AgNPs possess size-dependent interaction and the ability to block virus attachment and entry	[188]
8.	Respiratory syncytial virus	Pneumoviridae	Poly-vinylpyrolidone (PVP) coated silver nanospheres	8–12 nm	1 mg/mL and 2–4 mg/kg of mice	Procured from NanoComposix Inc. (San Diego, CA, USA)	AgNPs attached to surface glycol proteins and interfered with RSV’s ability to initiate attachment with the proper receptors, preventing fusion of the virus to the host cell	[189]
9.	Norovirus Surrogates		1 g/Kg	4–10 nm	21 mg/L	PHBV18 suspended in ultrapure Milli-Q water and then mixed with sodium borohydride and AgNO_3_	AgNPs reduce certain no. of NK cells	[190]
10.	Rhesus Rotavirus	Reoviridae	Collagen	10 nm	50 µL (0.4 mM)	The AgNP–collagen mixture gelled inside the abdominal cavity at temp. 37 °C	The virus load reduced in the liver due to increase in NK cells and T cells together	[191]
11.	Herpes Simplex Virus Type 2	Herpesviridae	Tannic-acid-modified AgNP	10–65 nm	2.5–5 µg/mL	Mixture of sodium citrate (4%) and tannic acid (5%) added to AgNO_3_ and stirred	AgNPs interact with the virion’s surface and create a physical obstacle, impairing interaction with the viral receptors on the cell surface	[192]
12.	Herpes Simplex Virus 2 (HSV-2)	Herpesviridae	Tannic-acid-modified AgNP	20–40 nm	5 µg/mL	Reducing agent added to aqueous solution of silver nitrate and heated to boiling point	Tannic acid has been shown to inhibit the attachment of viruses to host cells	[193]
13.	Zika Virus	Flaviviridae	* Rhazyastricta *	20–40 nm	5–120 µg/mL	Dried powdered leaf extract mixed with 1 mM AgNO_3_ at room temp.	Due to minute size, they effectively penetrate the infectious agent	[194]
14.	HIV-1	Retroviridae	* Rhizophoralamarckii *	12–28 nm	0.25–1 µg/mL	3 mL of extract reacted with 47 mL of 1 mM AgNO_3_ and incubated for 6 h	HIV-1 reverse transcriptase inhibitory activity	[195]
15.	H1N1 Influenzae	Orthomyxoviridae	Zanamivir AgNP	2–3 nm	2.5 µg/mL	Constant magnetic stirring for 30 min at room temp.	Zanamivir is neuraminidase (NA) inhibitor and binds with NA pocket to disturb enzyme reaction	[196]
16.	H1N1 Influenzae	Orthomyxoviridae	Oseltamivir	2–3 nm	2.5 μg/mL	0.1 mL Vit. C added to 4 mL AgNO_3_ at room temp.	Blocks the release of new virions from the cell’ smembrane and becomes resistant to the influenza A virus	[197]
17.	Respiratory Syncytial Virus (RSV)	Paramyxoviridae	* Curcuma longa *	0.23 nm	0.008–0.24 nM	Curcumin dissolved in DMSO and added to ultra-pure water. Vigorous stirring of AgNO_3_ (10 mM) at 100 °C	AgNPs could prevent the virus from entering into cells and its replication	[198]
18.	HSV-1, HAV-10, and CoxB4 virus	Herpesviridae	* Lampranthus* *coccineus *	10.12–27.89 nm	10–40 µg/mL	Aqueous extract added to 1 mM silver nitrate in the ratio 2:10 and kept in water bath for 10 min at 60 °C	Interacts with herpes simplex thymidine kinase, hepatitis A 3c proteinase and Coxsackie virus B4 3c protease	[199]
19.	HSV-1, HAV-10 andCoxB4 virus	Herpesviridae	* Malephora lutea F. Aizoaceae *	8.91–14.48 nm	10–40 µg/mL	Aqueous extract added to 1 mM silver nitrate in the ratio 2:10 and kept in water bath for 10 min at 60 °C	Interacts with herpes simplex thymidine kinase, hepatitis A 3c proteinase and Coxsackie virus B4 3c protease	[199]
20.	*Dengue Virus*	Flaviviridae	* Leucasaspera * * Hyptissuaveolens *	7–22 nm TEM 22–43 nm FRSEM	2–10 mg/L	2 mL of fresh extract added to 98 mL of aqueous silver nitrate (1 mM) solution and incubated at 28 °C for 60 min	The surface reactivity facilitated by capping makes these functionalized NPs a promising tool for vector control	[200]
21.	HIV-1	Retroviridae	PVP/BSA-coated AgNPs	1–10 nm	25 µg/mL	AgNPs procured from Nanotechnologies, Inc.	AgNPs bind with GP120 subunit of viral envelope glycoprotein	[201]
22.	Influenza	Orthomyxoviridae	Lipoic acid	8–12 nm	0.5–5 µg/mL	AgNPs procured from Nanocomposix company	AgNPs induced neutrophil and monocyte recruitment and increased the levels of KC (CXCL-1), IL-12 and IL-6, as soon as 4 h after AgNP injection	[202]
23.	Herpesvirus-1	Herpesviridae	Chemical reduction	15–50 nm	24 µg/mL	The solution prepared for two-fold serial dilutions with varied concentration	AgNPs at nontoxic concentrations were capable of inhibiting BoHV-1 when administered prior to viral infection	[203]
24.	HSV-1, HIV	Herpesviridae Retroviridae	Sonochemical method	1–10 nm	1–10 µmol/mL	Severe stirring at room temperature	Interact with viral envelope glycoprotein	[204]
25.	SARS-CoV-2	Coronaviridae	Polycotton AgNP-CS	10–28 nm	5%	NanoxTecnologia S.A.–São Carlos/SP-Brazil)	Binding of AgNPs with sulfur residues from the virus’s surface glycoproteins, preventing interaction with the receptor and its entry into the host cell	[205]
26.	H1N1 Influenza A Virus	Orthomyxoviridae	AgNP–chitosan	5.5–12.9 nm	62–77 µg/mL	Aqueous medium at room temperature	Virion and composite interacted as the NP size is very small and cause degradation of virus	[206]
27.	Dengue virus (DEN-2)	Flaviridae	*Moringa oleifera*	100 nm	20 µg/mL		Toxic action of *M. oleifera* AgNPs against *A. aegypti* may be linked to lectin content, which is able to affect digestive and detoxifying enzymes	[207]

## Data Availability

This paper does not report any new data.
